# LENS: A mammography-specific hybrid CNN-Transformer with lesion-aware evidence modeling

**DOI:** 10.1371/journal.pone.0350720

**Published:** 2026-09-01

**Authors:** Duc Quy Hoang, Van Kien Cao, Tan Nhu Nguyen, Ngoc Son Nguyen

**Affiliations:** 1 Faculty of Electronics Technology, Industrial University of Ho Chi Minh City, Ho Chi Minh City, Viet Nam; 2 Big Data Research for Infrastructure and Green Engineering (BRIDGE) Research Group, Ho Chi Minh City University of Transport, Ho Chi Minh City, Viet Nam; 3 School of Biomedical Engineering, International University, Ho Chi Minh City, Viet Nam; 4 Vietnam National University, Ho Chi Minh City, Viet Nam; King Abdulaziz University, SAUDI ARABIA

## Abstract

**Background:**

Artificial Intelligence (AI) models for mammography classification is prone to shortcut learning because diagnostically relevant evidence is typically sparse, localized, and easily dominated by non-lesion background context. This study aimed to develop a mammography-specific framework that integrates lesion-focused evidence with global image representations to improve classification performance and provide more clinically interpretable decision support.

**Methods:**

We propose LENS, a hybrid CNN-Transformer family designed specifically for mammography. LENS combines a lightweight multi-scale convolutional neural network (CNN) backbone with an alternating local-global Transformer encoder. In addition to global image representations, a weakly supervised lesion-aware branch identifies and aggregates suspicious regional evidence to support image-level prediction. LENS was evaluated on the large-scale VinDrMammo dataset for the three-class mammography task and compared to advanced architectures, including ConvNeXt, DINOv2, GMIC, and Swin Transformer under a unified experimental protocol.

**Results:**

LENS-Base achieved the highest overall Accuracy of 85.5%, Macro F1-score of 79.6%, and Matthews correlation coefficient of 0.65. Quantitative localization evaluation results indicated that the selected regional evidence frequently overlapped with annotated abnormalities. Furthermore, these promising results come with fewer parameters and lower FLOPs.

**Conclusion:**

LENS integrates local lesion-related features with global anatomical context to achieve improved class-balanced mammography classification while providing quantitatively supported lesion-focused evidence. These findings suggest that LENS is a promising framework for AI-assisted mammography screening.

## Introduction

The global incidence of breast cancer increases by 1−5% annually and remains one of the leading causes of malignancy-related mortality among women [1]. Mammography screening has been shown to reduce cancer mortality, underscoring its importance for early detection and improved outcomes [2–4]. Nevertheless, interpretation accuracy is influenced by reader expertise and workload pressures, causing inter-reader variability and inconsistent recall decisions [5,6]. Previous studies show that AI-supported systems can yield clinically competitive performance for breast cancer detection in retrospective or triage settings, suggesting value for improving interpretation and reducing radiologist workload when appropriately integrated into screening workflows [7–13]. Indeed, CNN-based systems have achieved considerable success in various mammography analysis tasks over the last decade due to their ability to automatically learn hierarchical features via convolutional layers and pooling operations [14–20]. Despite these advantages, CNNs are limited in their ability to capture long-range dependencies and global semantic information. Vision Transformers (ViTs) have addressed this limitation by leveraging self-attention mechanisms, enabling the modeling of relationships between distant image patches [21]. This ability is particularly useful in mammography interpretation, where subtle, spatially distributed indicators of malignancy might be overlooked by architectures limited to local receptive fields [15,22]. But the drawback of using a pure Transformer is that its self-attention module has quadratic computational complexity with respect to image size and struggles to extract local information. Recent advances introduce a hierarchical architecture with shifted-window attention [23], which has a much lower computational cost. Liu et al. [24] further enhance local window attention by introducing continuous position bias (CPB), a small multilayer perceptron (MLP) that maps log-spaced relative coordinates to per-head bias values, facilitating smooth interpolation and transferability. For more efficient spatial attention, Twins-SVT [25] introduced an alternating Local-Global attention mechanism that combines non-overlapping window self-attention (LSA) with Global Sub-sampled Attention (GSA). In GSA, each local window is sub-sampled to produce groups of representative tokens. All spatial tokens then cross-attend to this compact set of representatives, reducing the quadratic computational cost. The architecture also incorporates a Conditional Position Encoding Generator (PEG) [26], which replaces fixed absolute position embeddings with a lightweight depthwise convolution, allowing the model to flexibly handle different input resolutions.

On the other hand, a vast amount of work has aimed to design hybrid networks that combine ViT and CNNs to mitigate their respective weaknesses and leverage their complementary strengths [27–29]. This trend also naturally adapts to the medical images domain [30]. In the field of mammography analysis, Al-Tam et al. [31] and their subsequent work [32] trained a sequential hybrid CNN-ViT model on the region of interest (ROI) rather than the whole mammogram. Their remarkable results confirm the robustness of the hybrid network in the ROI-based classification problem. However, their works depend heavily on precise annotations, which are costly and not always available in clinical settings. Patheda et al. [33] designed a parallel hybrid architecture comprising a shallow CNNs branch that extracts fine-grained patterns and a ViT branch that models global interactions between image patches. Although their proposed model obtained performance comparable to that of CNN-based models, their study was conducted on a public Kaggle dataset and lacked peer review. Furthermore, computational efficiency analysis was absent. Another drawback is that these studies directly use vanilla ViT in their hybrid design, which is computationally intensive. Ahmed et al. [34] proposed a novel framework that integrated MaxViT as a model backbone, an evolutionary algorithm for optimal feature selection, and an XGBoost classifier. Their model reported 98.2% accuracy and an F1-score of 98.0% in the KAU-BCMD dataset, which is superior compared to other baselines. However, the KAU‑BCMD dataset is currently insufficient, making it difficult to reproduce the results. Authors in [35] introduced a novel hybrid framework that combines multi-scale local feature learning from InceptionNext blocks and global dependencies captured by a self-attention mechanism from Transformer blocks. Their model reported 98.2% accuracy in the mammography screening task. Recently, [36] proposed DeformNeXt-Swin, a staged hybrid CNN-Transformer framework for breast lesion classification. The framework synthesizes a deformable CNN and SwiGLU-GRN gate to improve morphology-sensitive local feature extraction in the early stage. The latter stage adapts hierarchical window attention to capture long-range semantic information. Their novel framework achieves an accuracy of 95.25% and an F1-score of 94.74% on mammography screening.

**Fig 1:**
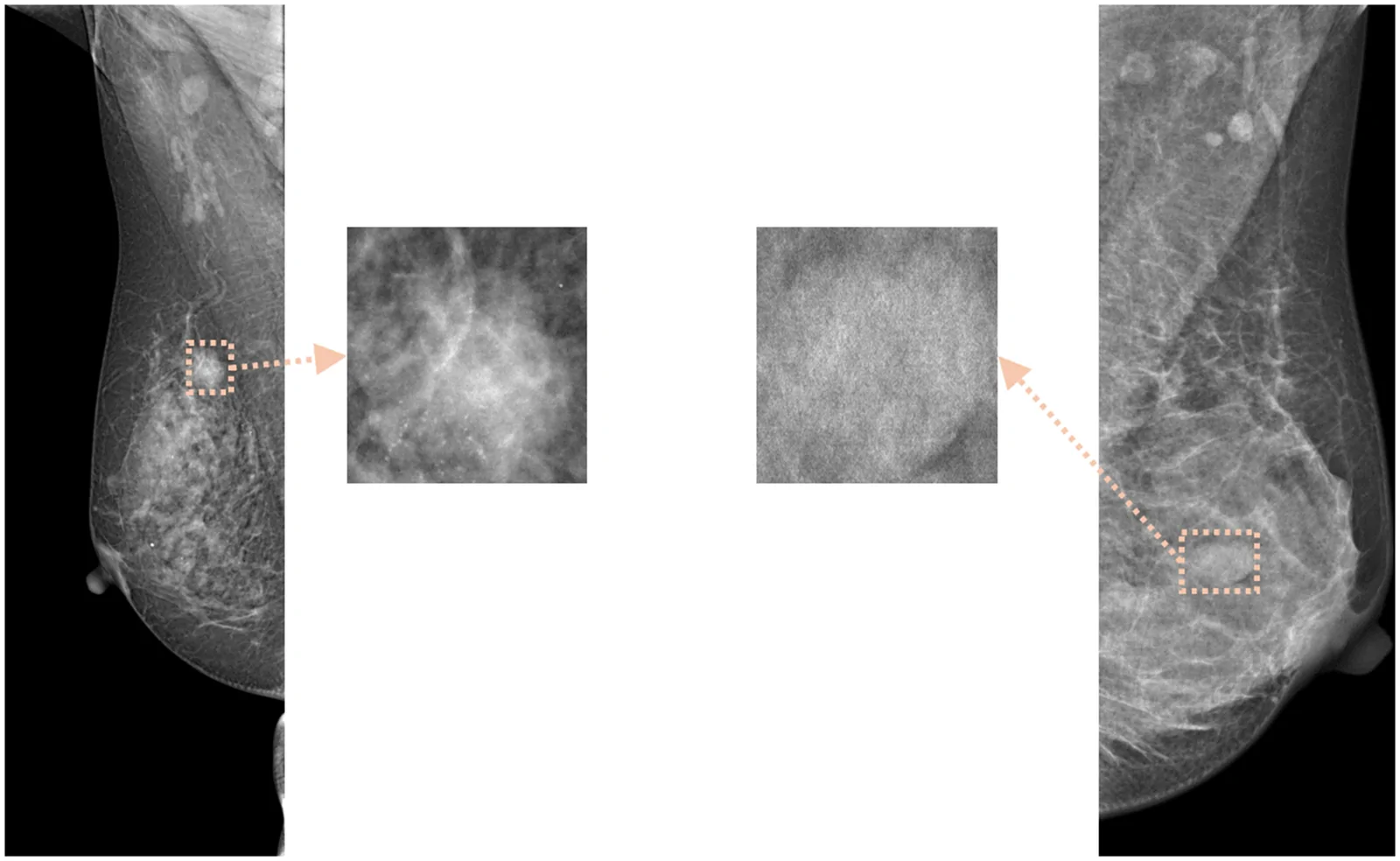
Example of mammograms and their annotated findings. Both show lesion evidence occupies only a small fraction of the image. In the left image, the lesion is clearly visible, whereas in the right image, it is less visible due to the masking effect of dense tissue.

In mammography analysis, a lesion typically occupies only a small fraction of the high-dimensional image (Fig 1), leading AI models to rely on acquisition artifacts, positioning patterns, and other non-lesion background cues, such as skin folds [16,37–39]. This problem is further complicated by class imbalance and lesion appearance. These challenges emphasize the need for advanced methods that are sensitive to small, spatially sparse lesions and can reason over broader anatomical contexts. For example, vast studies have proposed anchoring mammography classification in localized evidence by adopting Multiple-instance learning (MIL) [15,22,38,40]. Zhu et al. [15] and Hu et al. [40] ranked the likelihood of malignancy for regions on the CNN’s feature map using only the image-level label. Shen et al. [38] introduced a global-aware multiple instance classifier (GMIC), which combines a low-capacity global network for identifying informative regions with a high-capacity local network for detailed analysis. However, gradient flow is applied at the region-selection step, so the global and local branches optimize in a decoupled manner. To this end, Sun et al. [22] proposed a multi-scale region selection network (MRSN) in deep features. The model performs two rounds of region selection: channel-wise to identify feature types meaningful for malignancy and spatial-wise to locate tumor regions. Finally, they fused the top-K regions from each branch before prediction. Despite achieving promising results, their simple aggregation strategies, such as average pooling, may dilute important features. [41] introduced the FPN-MIL model for breast cancer classification and detection in high-resolution mammograms, comprising: an FPN-based instance encoder that performs multi-scale feature extraction on input patches, attention-driven instance aggregators, and an attention-driven multi-scale aggregator that adaptively weighted and fused scale-specific features, enhancing robustness to lesion variability.

Based on the above observations, we propose a hybrid deep learning framework with lesion-aware evidence modeling. The framework combines a CNN-based multi-scale feature fusion (MSFF) backbone with a Transformer-based alternating local and global attention block, enabling complementary strengths while maintaining an acceptable computational cost. In addition to global representation learning, we introduce a lesion-aware branch trained solely on image-level supervision. Unlike GMIC [38] and MRSN [22], LENS retains end-to-end differentiability by integrating lesion-aware evidence routing directly into the forward computation. Furthermore, while GMIC and MRSN rely on separate or staged pathways for region processing, LENS provides direct, contextual refinement of the top-k suspicious regions with dedicated self-attention, guaranteeing that key suspicious evidence is dynamically informed by the overall image context. This results in improved accuracy and explainability, granting a more effective and interpretable mammography screening solution. The task is formulated as a three-class mammography screening problem with labels: Normal (BI-RADS 1), Benign (BI-RADS 2), and Recall (BI-RADS 4–5). The primary contributions of this work are:

(1) An end-to-end lesion-aware evidence-routing mechanism that scores token-level lesion evidence from image-level supervision, contextually refines the top-k suspicious tokens, and integrates the resulting lesion representation directly into the final classification pathway.
(2) A mammography-specific hybrid CNN-Transformer architecture that uses multi-scale rectangular tokenization to preserve the elongated geometry of mammograms and local-global contextual modeling to integrate local features with broader anatomical context
(3) A systematic empirical evaluation, including comparisons with seven CNN, Transformer, and mammography-specific baselines under a unified experimental protocol, detailed ablation studies, quantitative lesion-localization analysis against radiologist annotations, and computational-efficiency profiling.

The remainder of the paper provides a detailed description of our LENS architecture in the Methods section. The Results section reports comprehensive quantitative findings that highlight the effectiveness of our approach. In the Discussion section, we reflect on our contributions, limitations, and directions for future research, and we conclude with a summary in the Conclusion section.

## Methods

### Dataset and preprocessing

We use the large-scale public dataset with 5000 exams (20000 images), namely VinDr-Mammo [42], which provides breast-level BI-RADS assessments for FFDM images and lesion-level annotations for a subset of abnormal findings. The dataset was acquired retrospectively from two primary hospitals in Vietnam, namely Hospital 108 (H108) and Hanoi Medical University Hospital (HMUH). The label assignment follows Breast Imaging Reporting and Data Systems (BI-RADS) [43]. In this study, we include only cases with BI-RADS 1, 2, 4, and 5 assessments. Specifically, we convert the provided BI-RADS labels into a 3-class screening task: Normal (BI-RADS 1), Benign (BI-RADS 2), and Recall (BI-RADS 4 or 5). BI-RADS 4 and 5 both result in the same immediate clinical action (recall for biopsy or additional imaging). From a screening workflow perspective, the distinction between “does not require recall” and “requires recall” is the operationally relevant decision. We merge these categories to reflect this decision boundary. Meanwhile, BI-RADS 3 cases are excluded because, in clinical practice, BI-RADS 3 is defined as “probably benign” and recommends short-interval follow-up rather than immediate recall. On the other hand, merging BI-RADS 3 with the benign class may increase the risk of false negatives; therefore, it is not recommended in screening [44] without comprehensive diagnostic workup [45]. This exclusion is also consistent with prior computational screening studies on VinDr-Mammo [46,47] and reflects the clinical reality that the critical triage decision is between routine screening (BI-RADS 1, 2) and recall for further assessment (BI-RADS 4, 5). The details of the dataset after excluding BI-RADS 3 are shown in Table 1. The dataset split follows the official protocol described in [42], with disjoint training (80%) and test (20%) subsets. These splits are performed at the patient level to prevent data leakage and guarantee that no test data is used at any stage of model development.

**Table 1:** VinDrMammo dataset after removing BI-RADS 3 cases. Each cell presents the number of images and the distribution of each class.

	Split		
Category	Training	Test	Overall
Normal	10,725 (70.3%)	2,681 (70.3%)	13,406 (70.3%)
Benign	3,741 (24.5%)	935 (24.5%)	4,676 (24.5%)
Recall	790 (5.2%)	198 (5.2%)	988 (5.2%)
Total	15,256	3,814	19,070

FFDM images contain large background regions and acquisition-specific artifacts outside the breast area, as illustrated in Fig 1. Therefore, we adopt the breast-region cropping method from our previous work [48] to remove irrelevant background. All images are resized to 1024×512 that respects the natural ratio of mammograms. During the data-loading phase of model training, we apply image transformation techniques to augment the training data and improve model robustness to real-world variations in mammographic images. Specifically, we employ the following augmentations: random rotation within ±10 degrees (prob = 0.5), Gaussian noise injection with a standard deviation of 0.05 (prob = 0.5), and random scaling between 0.9 and 1.1 (prob = 0.3). All augmentation parameters were empirically selected to simulate common image acquisition and patient positioning variabilities.

### LENS architecture

**Fig 2:**
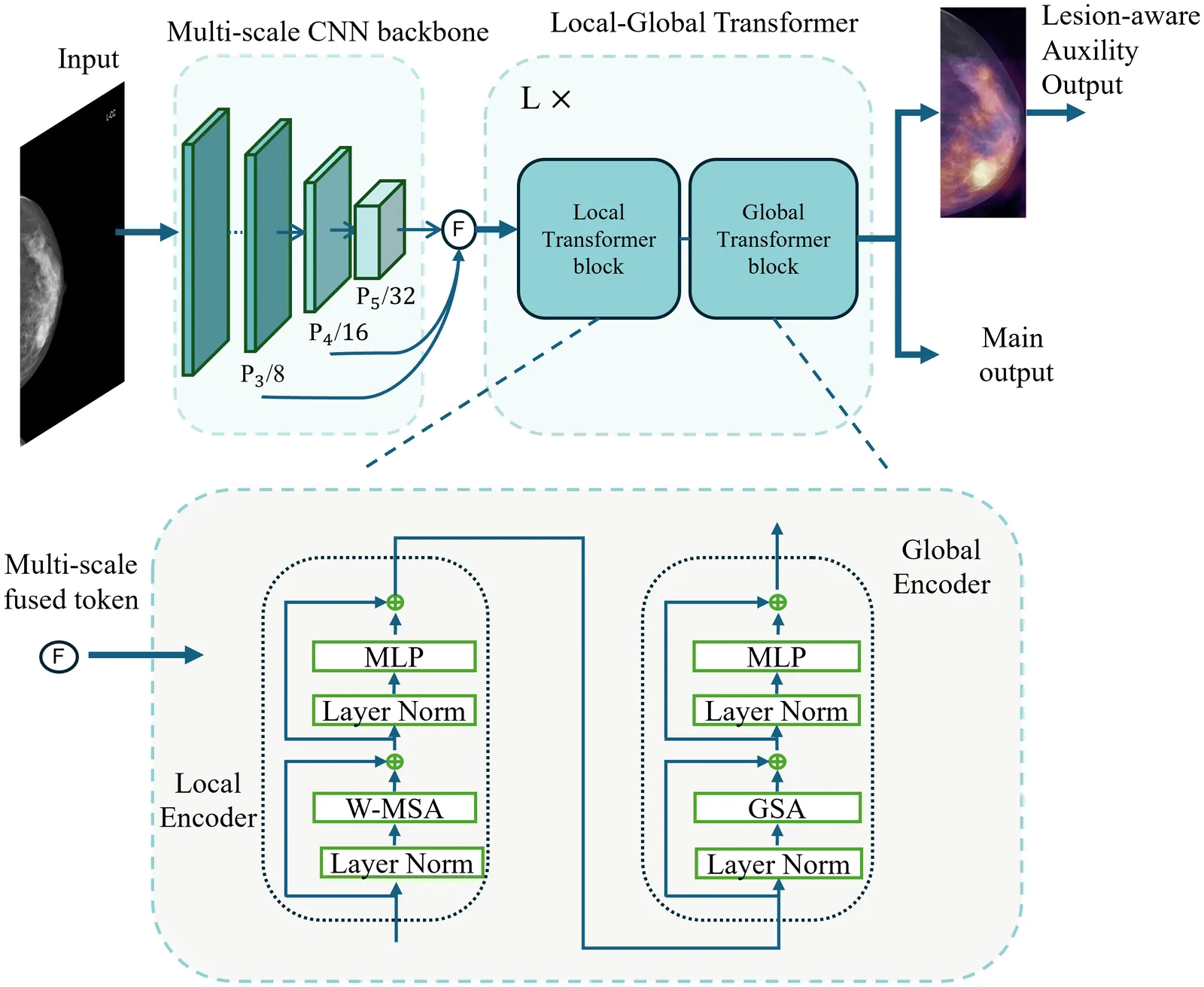
Overview of LENS architecture.

Fig 2 summarizes our proposed architecture. It integrates an EfficientNet-inspired [49,50] multi-scale feature fusion (MSFF) backbone with a geometry-aware local-global Transformer encoder and produces an explicit lesion-aware evidence branch. The design is motivated by two characteristics of mammography classification: diagnostically relevant structure is spatially sparse relative to the full field of view, yet correct classification also depends on large-scale anatomical context. Accordingly, the model is designed to preserve both long-axis breast geometry and a dedicated pathway for localized suspicious evidence.

### Multi-scale feature fusion backbone

Let $x\in {\mathbb{R}}^{H_{0}\times W_{0}\times 1}$ denote a cropped mammogram. The MSFF backbone produces a three-level feature pyramid $\left\{P_{3},P_{4},P_{5}\right\}$, where $P_{s}\in {\mathbb{R}}^{H_{s}\times W_{s}\times C_{s}}$ denotes the feature map at stage *s*. Each stage is projected to a common Transformer token dimension *d* by a 1×1 convolution $\phi_{s}(\cdot )$ and resized to a shared grid $\left(H_{g}=\frac{H_{0}}{32},W_{g}=\frac{W_{0}}{32}\right)$ using bilinear interpolation,


$$\begin{array}{c} {\tilde{P}}_{s}=ℛ(\phi_{s}(P_{s})),\;s\in \{3,4,5\}, \end{array}$$ (1)

where ${\tilde{P}}_{s}\in {\mathbb{R}}^{H_{g}\times W_{g}\times d}$ in Eqs.1 is tokenized feature at statge s. Rather than using a fixed, unweighted sum or average, LENS adopts learnable, softmax-based scale-weight fusion [50].


$$\begin{array}{c} F=\sum_{s=3}^{5}\pi_{s}{\tilde{P}}_{s},\;\pi_{s}=\frac{\exp(a_{s})}{\sum_{t=3}^{5}\exp(a_{t})}, \end{array}$$ (2)

where $a_{s}$ are trainable fusion coefficients. This formulation allows the model to adaptively harmonize fine-grained and coarse semantic evidence during training.

### Transformer encoder

Given input token sequence $X^{\left(\ell -1\right)}$, each Transformer block follows a pre-norm residual local-global update,


$$\begin{array}{ccc} {\hat{Z}}^{\left(\ell \right)} & = & X^{\left(\ell -1\right)}+{𝒜}_{L}(LN(X^{\left(\ell -1\right)})), \end{array}$$ (3)

$$\begin{array}{ccc} Z^{\left(\ell \right)} & = & {\hat{X}}^{\left(\ell \right)}+{MLP}_{\ell }(LN({\hat{Z}}^{\left(\ell \right)})), \end{array}$$ (4)

$$\begin{array}{ccc} {\hat{X}}^{\left(\ell +1\right)} & = & {\hat{Z}}^{\left(\ell \right)}+{𝒜}_{G}(LN({\hat{Z}}^{\left(\ell \right)})), \end{array}$$ (5)

$$\begin{array}{ccc} X^{\left(\ell +1\right)} & = & {\hat{X}}^{\left(\ell +1\right)}+{MLP}_{\ell }(LN({\hat{X}}^{\left(\ell +1\right)})), \end{array}$$ (6)

where ${𝒜}_{L}$ denotes the non-overlapping window attention operator, ${𝒜}_{G}$ denotes the global attention at block ℓ. For the LENS-small configuration, the encoder uses *L* = 6 consecutive LG blocks with embedding dimension *d* = 384 and 6 attention heads. Table 2 shows the detailed configuration of the proposed architecture. Overall, we introduce 3 variants: LENS-tiny, LENS-small, and LENS-base.

**Table 2:** Architectures of the proposed LENS variants for mammography classification. All variants use the same EfficientNet-B3 backbone and a shared hybrid local-global encoder, differing only in the embedding dimension *d*, the number of attention heads *H*, and the expansion ratio in the MLP layer. Computational complexity is reported for an input size of 1024×512.

Stage	Output Size	LENS-T	LENS-S	LENS-B
*P*_1_	512×256	Output feature map at stage 1		
*P*_2_	256×128	Output feature map at stage 2		
*P*_3_	128×64	Output feature map at stage 3		
*P*_4_	64×32	Output feature map at stage 4		
*P*_5_	32×16	Output feature map at stage 5		
MSFF	32×16×d	Multi-scale fusion in Eqs.2		
LG blocks	32×16×d	$\left[\begin{array}{c} H=4,\\ d=256\\ R=4 \end{array}\right]\times 2$	$\left[\begin{array}{c} H=6,\\ d=384\\ R=4 \end{array}\right]\times 6$	$\left[\begin{array}{c} H=8,\\ d=512\\ R=4 \end{array}\right]\times 9$
Head	Fully Connected Layer, 3			
Params		14.93M	26.99M	48.79M
FLOPs		22.42G	31.51G	48.56G

### Local window block

Let $X^{\left(\ell -1\right)}=[x_{cls};z_{1},...,z_{N}]\in {\mathbb{R}}^{(1+N)\times d}$ denote the input token sequence at block ℓ. The local block partitions the $N=H_{g}W_{g}$ spatial tokens into $n_{w}=\frac{H_{g}}{w}\cdot \frac{W_{g}}{w}$ non-overlapping windows of size w×w, collecting the tokens of window *j* into a matrix $W_{j}\in {\mathbb{R}}^{w^{2}\times d}$. To allow the classification token to aggregate spatial context from all windows in parallel, $x_{cls}$ is broadcast into every window before attention. Define the augmented window sequence


$$\begin{array}{c} {\tilde{W}}_{j}=[x_{cls};\;W_{j}]\in {\mathbb{R}}^{(1+w^{2})\times d},\;j=1,...,n_{w}. \end{array}$$ (7)

Window-level self-attention with a continuous position bias (CPB) [24] is applied independently to each ${\tilde{W}}_{j}$,


$$\begin{array}{c} {\tilde{W}}_{j}^{\prime }=Softmax(QK^{T}/\sqrt{d}+B_{j})V, \end{array}$$ (8)

where $B_{j}\in {\mathbb{R}}^{H\times (1+w^{2})\times (1+w^{2})}$ is the per-head additive CPB matrix. The CPB is produced by a two-layer MLP operating on log-spaced normalized relative 2-D coordinates. The CLS outputs of all windows are averaged to form a single updated CLS token,


$$\begin{array}{c} x_{cls}^{\prime }=\frac{1}{n_{w}}\sum_{j=1}^{n_{w}}{\tilde{W}}_{j}^{\prime }[\;0\;], \end{array}$$ (9)

while the spatial outputs are merged back to the original grid layout. The full pre-norm residual update then reads as in Eqs. 3 and 4.

### Global sub-sampled block

The global block replaces full $𝒪(N^{2})$ self-attention with the Sub-sampled Attention (GSA) strategy of Twins-SVT [25]. Different from them, each window $W_{j}$ is reduced to a single representative token by average pooling,


$$\begin{array}{c} r_{j}=\frac{1}{w^{2}}\sum_{i\in W_{j}}z_{i},\;j=1,...,n_{w}, \end{array}$$ (10)

yielding a compact summary sequence $R=[r_{1},...,r_{n_{w}}]\in {\mathbb{R}}^{n_{w}\times d}$ that is layer-normalized before serving as keys and values. Cross-attention is then computed with *Q* drawn from the input sequence token $Z\in {\mathbb{R}}^{(1+N)\times d}$ and *K*, *V* drawn from *R*, which reduces the attention complexity from $𝒪(N^{2})$ to $𝒪(N\cdot n_{w})$. For the default configuration ($H_{g}=32$, $W_{g}=16$, *w* = 8), $n_{w}=8$ versus *N* = 512, yielding a 64× reduction in key/value tokens.

### Lesion-aware evidence modeling

LENS complements the global evidence branch with a lesion-aware branch that explicitly focuses on suspicious local regions. Given spatial tokens $\{z_{i}{\}}_{i=1}^{N}$, a lightweight scoring MLP estimates the lesion relevance of each token, denoted as $f_{score}$, and converts them into a soft evidence distribution in Eqs.11,


$$\begin{array}{c} s_{i}=f_{score}(z_{i}),\;\omega_{i}=\frac{\exp(s_{i}/T)}{\sum_{j=1}^{N}\exp(s_{j}/T)}, \end{array}$$ (11)

where *T* = 0.1 is a lesion-score temperature hyperparameter that controls how sharp the lesion-evidence distribution is. Each token is then modulated by a channel-wise sigmoid gate with residual scaling α,


$$\begin{array}{c} {\tilde{z}}_{i}=z_{i}⊙\left(1+\alpha \;\sigma \;\left(f_{gate}(z_{i})\right)\right), \end{array}$$ (12)

where the gate assigns an importance value in [0,1] to each feature channel, and α=0.5 controls how strongly the gate amplifies lesion candidate tokens. A soft lesion summary is obtained via weighted aggregation,


$$\begin{array}{c} {\bar{z}}_{les}=\sum_{i=1}^{N}\omega_{i}{\tilde{z}}_{i} \end{array}$$ (13)

To preserve sparse but highly confident evidence, we select the top-k (*k* = 8) tokens with the highest lesion scores. The ablation study section demonstrated the impact of the top-k value empirically. We then combine them with a learnable lesion token, the classification token and refine this compact token set using a small stack of lesion-refinement blocks. The refined lesion representation $z_{ref}$ is then fused with the global class token $z_{0}\in Z$ and the soft lesion summary in Eqs.13 for the final prediction.


$$\begin{array}{c} h=concat[z_{0},\|\;{\bar{z}}_{les}\;\|\;z_{ref}],\;\hat{y}=f_{cls}(h), \end{array}$$ (14)

where $f_{cls}(h)$ is a two-layer MLP. An optional auxiliary classifier on lesion-branch features, denoted as $f_{cls}({\bar{z}}_{les})$, can be used during training to provide additional supervision. Unlike post-hoc saliency methods [22,38], the mined lesion evidence directly participates in the forward prediction process. We trained the model end-to-end with the following loss function:


$$\begin{array}{c} {ℒ}_{\left(y,\hat{y}\right)}={ℒ}_{main}(y,f_{cls}(h))+\lambda {ℒ}_{aux}(y,f_{cls}({\bar{z}}_{les}), \end{array}$$ (15)

where ${ℒ}_{main}$ and ${ℒ}_{aux}$ is the focal loss [51] for main classification head and auxiliary head respectively, λ is auxiliary loss weight.

## Results

### Experimental settings

#### Cross-validation settings.

5-fold stratified cross-validation was performed on the training set only (see Table 1), ensuring each fold maintains the clinical distribution of the dataset. The fold that achieved the highest validation Macro-F1 was selected to identify the best model checkpoint for that architecture and was used to evaluate on the held-out test set.

#### Implementation settings.

LENS and all comparator baselines were trained, validated, and evaluated under the same protocol: identical patient-level data splits, image preprocessing, augmentation pipeline, class weighting, optimizer and scheduler configuration, fold-selection criterion (best validation Macro-F1), and test-set evaluation. Specifically, all models were trained for a 3-class screening task using focal loss with weights proportional to inverse class frequency and γ=2 to mitigate class imbalance. We trained each model for 100 epochs using the AdamW [52] optimizer, with the learning rate linearly increased from 1e−8 to 1e−4 in 5 warmup epochs, then gradually decreased using cosine annealing decay, weight decay of 0.05, dropout of 0.5 for all MLP layers, and a drop-path rate of 0.2 for all Transformer blocks. All Experiments are implemented in PyTorch [53] and conducted on an NVIDIA A6000 GPU. A summary of key hyperparameters of LENS is presented explicitly in Table 3.

**Table 3:** Key hyperparameters of the proposed model.

Hyperparameter	Value/ State
Top-*k* lesion tokens	8
Lesion temperature (*T*)	0.1
Residual scaling (α)	0.5
Lesion-refinement depth	2
Window size	8×8
Focal loss class weight	[0.16, 0.26, 0.58]
Focal loss γ	2
Auxiliary head	Enable
Auxiliary weight	0.4

### Statistical analysis

We report overall Accuracy, AUC, macro F1-score, and the Matthews Correlation Coefficient (MCC) to account for class imbalance and operating-point robustness. In addition to these aggregate metrics, per-class Sensitivity, Specificity, Positive Predictive Value (PPV), and Negative Predictive Value (NPV) are used to characterize class-specific operating behavior for the Normal, Benign, and Recall categories. We also report sensitivity at a false-positive rate of 10%, denoted as Sen@FPR10, as a screening-relevant measure of the sensitivity and false-positive rate trade-off. Confusion matrices and per-class ROC curves are presented for LENS variants to validate per-class performance at different scales. For statistical analysis, we estimated paired-bootstrap 95% confidence intervals for the performance difference using 10,000 resamples. We tested statistical differences in Macro-F1, Macro-AUC, and MCC using two-sided paired permutation tests and the McNemar test for accuracy.

The VinDr-Mammo dataset does include ground-truth (GT) bounding boxes for a subset of abnormal cases. We have used these annotations to quantify the localization performance of the lesion-aware evidence branch. It is worth noting that LENS is designed to mine multiple discriminative lesion tokens for classification rather than to regress a single lesion bounding box. Accordingly, we report token-alignment metrics as the primary localization analysis. We use Pointing Game accuracy [54], Top-k token hit rate, Top-k token union IoU, and Heatmap Coverage of GT. For Top-k token hit rate, we select the highest-scoring lesion tokens from the lesion score map (*k* = 8 in our experiments). A case is counted as a hit when at least one selected token spatially overlaps any radiologist-annotated GT bounding box. For Top-k token union IoU (Top-k IoU), each selected token is converted to its corresponding image-space cell, and the union of the *k* selected cells is compared with GT bounding-box regions. In addition, we report Heatmap Coverage of GT to quantify whether the lesion score map assigns elevated activation within annotated lesion regions. A token is considered GT-overlapping if its spatial cell intersects at least one GT bounding box. We first project each GT box onto the lesion-token grid H×W. For an image *i*, we compute the mean lesion-token score ${\bar{s}}_{i}$ over the full grid, then calculate the fraction of GT-overlapping token cells whose score exceeds this image-level mean:


$$\begin{array}{c} {Heatmap\;Coverage}_{i}=\frac{\sum_{t\in {𝒢}_{i}^{grid}}1[s_{i,t}>{\bar{s}}_{i}]}{\left|{𝒢}_{i}^{grid}\right|}, \end{array}$$ (16)

where $\left|{𝒢}_{i}^{grid}\right|$ is the number of ground-truth-overlapping token cells for image *i*, and $s_{i,t}$ is the lesion score of token *t*. A high Heatmap Coverage value indicates that a large proportion of clinically annotated lesion cells receive high lesion ac*t*ivation.

### Mammography screening results

Table 4 presents a comprehensive comparison between LENS and other state-of-the-art baselines: the advanced CNN-based model ConvNeXt [55], the foundation ViT-based DINOv2 [56], the local attention-based model Swin Transformer [23], and the mammography-specific model GMIC [38] on the VinDr-Mammo test set. Overall, LENS-B achieved the strongest performance across most aggregate metrics. Specifically, it achieved the highest Accuracy of 85.5% (95% CI: 84.2–86.7), Macro-F1 of 79.6% (95% CI: 77.7–81.4), and MCC of 0.65 (95% CI:0.63–0.68). Meanwhile, Swin-B obtained the highest Macro AUC at 90.2% (95% CI: 88.9–91.4), whereas LENS-B reached 87.6% (95% CI: 86.2–89.1).

**Table 4:** Architecture comparison on the VinDr-Mammo test set. Values are reported as mean with bootstrap 95% confidence intervals.

Model	Accuracy (%)	Macro-F1 (%)	Macro AUC (%)	MCC
ConvNeXt-B [55]	83.8 [82.7, 85.0]	76.5 [74.3, 78.4]	88.4 [87.0, 89.8]	0.62 [0.59, 0.64]
ConvNeXt-S [55]	82.5 [81.2, 83.6]	74.2 [71.8, 76.3]	86.3 [84.8, 87.7]	0.59 [0.56, 0.62]
DINOv2-B [56]	81.8 [80.7, 83.0]	69.4 [67.0, 71.8]	83.7 [82.3, 85.4]	0.56 [0.54, 0.59]
DINOv2-S [56]	81.7 [80.4, 83.0]	69.3 [67.0, 71.5]	83.7 [82.5, 85.0]	0.57 [0.54, 0.59]
Swin-B [23]	80.1 [78.8, 81.3]	75.2 [73.1, 77.3]	**90.2** [88.9, 91.4]	0.60 [0.58, 0.63]
Swin-S [23]	81.5 [80.2, 82.7]	69.6 [67.3, 71.8]	86.7 [85.4, 88.0]	0.58 [0.56, 0.61]
GMIC [38]	85.1 [83.9, 86.4]	75.5 [72.8, 77.8]	87.8 [86.3, 89.3]	0.64 [0.62, 0.67]
**LENS-B**	**85.5** [84.2, 86.7]	**79.6** [77.7, 81.4]	87.6 [86.2, 89.1]	**0.65** [0.63, 0.68]
**LENS-S**	83.9 [82.8, 85.1]	77.5 [75.4, 79.4]	85.7 [83.9, 87.4]	0.63 [0.60, 0.66]
**LENS-T**	80.2 [78.9, 81.4]	68.1 [65.8, 70.2]	83.9 [82.4, 85.4]	0.54 [0.51, 0.57]

**Fig 3:**
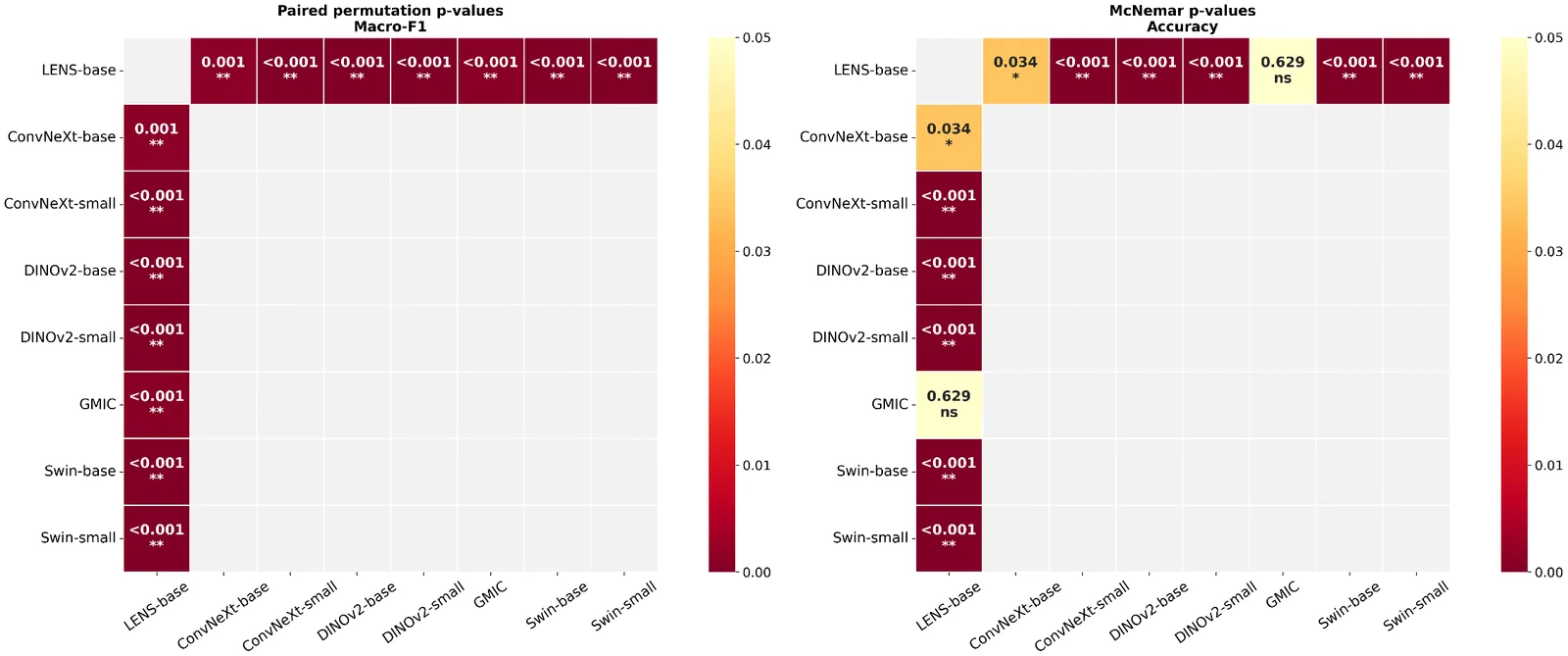
Statistical test heatmap on F1-score and Accuracy. * denotes p-values < 0.05, ** denotes p-values <0.001, and ns denotes non-significance (p-values > 0.05).

Moreover, pairwise statistical testing (Fig 3) confirms that the improvement in Macro-F1 is consistent across the evaluated baselines. Specifically, two-sided paired permutation tests show that LENS-B significantly outperforms ConvNeXt-B (*p* = 0.001) and all other baseline models (*p* < 0.001) in Macro-F1. For Accuracy, the McNemar test indicates a significant difference between LENS-B and ConvNeXt-B (*p* = 0.034), as well as between LENS-B and ConvNeXt-S, DINOv2-B, DINOv2-S, Swin-B, and Swin-S (all *p* < 0.001). In contrast, the Accuracy difference between LENS-B and GMIC is not statistically significant (*p* > 0.05), indicating comparable overall classification accuracy between these two models. On the other hand, the differences in Macro-AUC and MCC are not statistically significant (*p* > 0.05). Thus, although Swin-B has the highest Macro-AUC and LENS-B has the highest MCC, these differences are not supported by the corresponding paired statistical tests. Finally, the scaling behavior of LENS variants further supports the effectiveness of the proposed design. While LENS-S maintains competitive performance close to LENS-B, LENS-T shows a noticeable degradation, particularly in Macro-F1 (68.1%) and MCC (0.54). Nevertheless, LENS-T remains comparable to strong baselines such as DINOv2, indicating that the proposed architecture degrades gracefully under reduced model capacity.

Table 5 reports class-specific operating-point metrics for the Normal, Benign, and Recall categories. LENS-B provides a balanced profile across classes, with high Normal sensitivity (94.2%) and strong performance for the clinically important Recall category, including the highest Recall PPV (92.7%) and specificity (99.7%). Compared with GMIC, LENS-B substantially increased Recall sensitivity from 52.0% to 70.6% while also maintaining higher Recall specificity and PPV. For the Benign category, Swin-B achieved the highest sensitivity of 72.0%, but this was accompanied by a lower specificity of 81.3% and a PPV of 59.3%, indicating a greater false-positive burden. GMIC achieved higher Benign sensitivity than LENS-B (65.1% vs. 61.7%), whereas LENS-B provided slightly higher PPV and NPV. The results across the LENS variants also show a reduction in performance when decreasing model capacity. At a fixed false-positive rate of 10%, LENS-B achieved sensitivities of 65.8%, 70.9%, and 82.8% for the Normal, Benign, and Recall classes, respectively. It obtained the highest Sen@FPR10 for both the Normal and Recall categories, while its Benign result was close to the highest value achieved by GMIC (70.9% vs. 71.2%). Overall, LENS-B provided the most favorable balance between sensitivity, specificity, and precision for Recall predictions while maintaining comparative performance across the remaining screening categories.

**Table 5:** Per-class operating-point metrics on the VinDr-Mammo baseline evaluation. Entries are percentages in the order Normal / Benign / Recall. 95% confidence intervals are removed for readability.

Model	Sensitivity	Specificity	NPV	PPV	Sen@FPR10
ConvNeXt-B	93.7 / 57.9 / 74.2	65.5 / 94.0 / 98.6	81.4 / 87.3 / 98.6	86.5 / 75.9 / 73.9	59.5 / 65.8 / 79.3
ConvNeXt-S	91.2 / 61.0 / 65.7	66.8 / 91.7 / 98.6	76.3 / 87.8 / 98.1	86.7 / 70.4 / 71.4	49.3 / 62.6 / 80.3
DINOv2-B	97.8 / 42.1 / 53.5	48.6 / 97.3 / 99.1	90.5 / 83.8 / 97.5	81.8 / 83.7 / 76.8	39.8 / 54.9 / 73.2
DINOv2-S	94.9 / 47.1 / 67.2	59.7 / 95.6 / 96.9	83.2 / 84.8 / 98.2	84.8 / 77.7 / 54.1	35.5 / 56.8 / 76.3
GMIC	94.8 / 65.1 / 52.0	67.1 / 93.8 / 99.1	82.4 / 87.2 / 97.4	86.2 / 75.4 / 91.1	55.5 / 71.2 / 77.8
Swin-B	80.9 / 72.0 / 71.3	80.0 / 81.3 / 98.2	65.0 / 92.4 / 98.1	92.3 / 59.3 / 69.3	57.3 / 65.3 / 81.3
Swin-S	90.0 / 58.9 / 71.7	71.4 / 92.7 / 95.2	75.2 / 87.4 / 98.4	88.2 / 72.5 / 44.9	56.0 / 64.9 / 76.3
LENS-B	94.2 / 61.7 / 70.7	66.1 / 93.8 / 99.7	82.8 / 88.3 / 98.4	86.8 / 76.5 / 92.7	65.8 / 70.9 / 82.8
LENS-S	93.8 / 57.9 / 74.2	64.7 / 94.0 / 99.0	81.5 / 87.3 / 98.6	86.3 / 75.7 / 79.9	45.4 / 63.5 / 79.3
LENS-T	91.0 / 51.3 / 70.2	63.2 / 93.8 / 95.6	74.7 / 85.6 / 98.3	85.4 / 72.8 / 46.5	42.0 / 57.3 / 78.3

Table 6 summarizes the computational efficiency of all models under a unified hardware setting and input resolution (1024×512). For the throughput, models perform inference with a batch size of 4. Overall, the proposed LENS family demonstrates a favorable trade-off between computational cost and inference speed. Smaller variants such as LENS-T and LENS-S achieve very low complexity (22.42G and 31.51G FLOPs, respectively) while maintaining high throughput (136 and 120 img/s). Importantly, LENS-B scales to 48.79M parameters and 48.56G FLOPs while still delivering 102 img/s, substantially outperforming comparable-sized architectures. For instance, ConvNeXt-S and Swin-S require approximately 4× higher FLOPs and achieve only 52 and 33 img/s, respectively. The efficiency gap is more pronounced than with pure Transformer models. DINOv2-B incurs 703.85G FLOPs and the highest memory footprint (1252.08 MB), resulting in a low throughput of 13 img/s. In contrast, LENS-B reduces FLOPs by over 14× and nearly halves memory usage, while improving throughput by approximately 7.5×. Compared with the efficient GMIC, which has the highest throughput (150 img/s), LENS-B uses ~49% fewer FLOPs while retaining over 67% of the throughput.

**Table 6:** Efficiency profiling of the LENS family and baselines. Model parameters, floating-point operations (FLOPs), Throughput, and peak GPU memory are measured under the same hardware configuration as the training setting. Input size is 1024×512.

Model	Params (M)	FLOPs (G)	Throughput (img/s)	Memory (MB)
ConvNeXt-S	49.46	181.50	52	536.66
ConvNeXt-B	87.57	320.92	36	721.16
DINOv2-S	22.06	240.28	31	659.60
DINOv2-B	86.59	703.85	13	1252.08
Swin-S	48.84	195.07	33	627.65
Swin-B	86.75	344.15	23	840.20
GMIC	32.60	95.32	150	416.86
**LENS-T**	14.93	22.42	136	443.65
**LENS-S**	26.99	31.51	120	489.90
**LENS-B**	48.79	48.56	102	574.92

**Fig 4:**
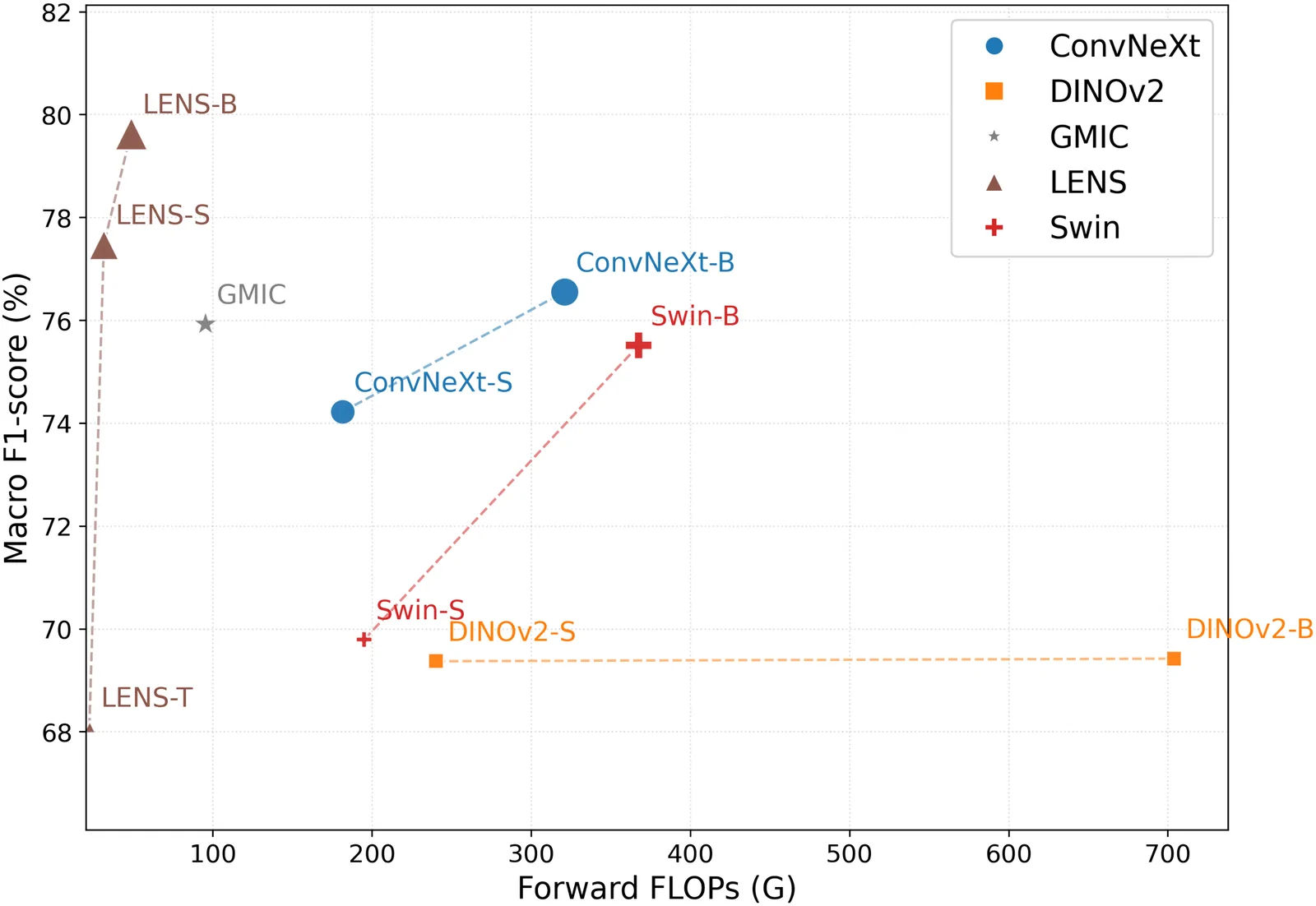
FLOPs vs F1-score comparison plot. LENS achieves significantly higher performance while maintaining a lower computational cost than other advanced architectures.

**Fig 5:**
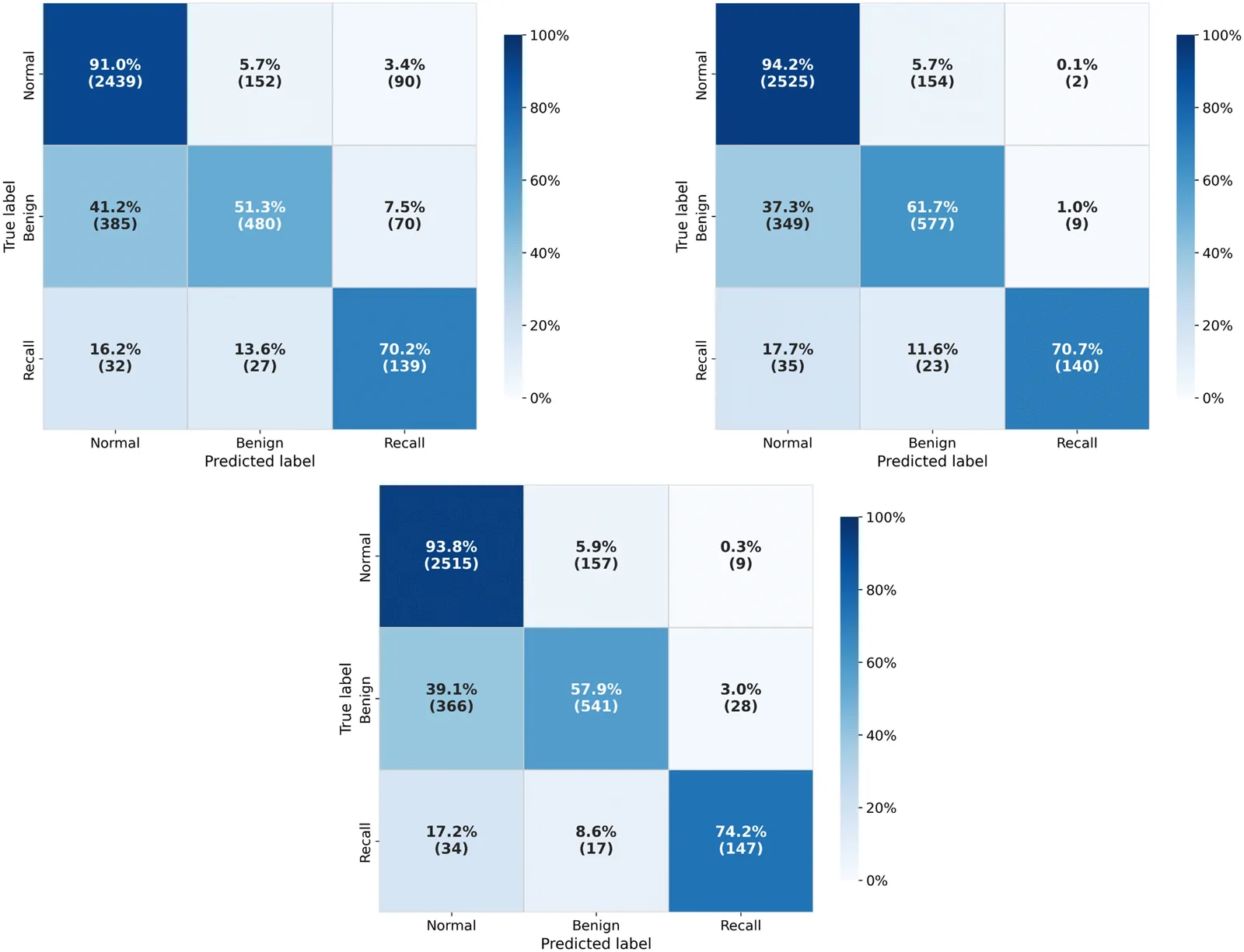
Confusion matrices for LENS-variant. The first row presents LENS-Tiny (left) and LENS-Small (right); The second row presents LENS-Base. All models classify the Normal class reliably, while the main remaining confusion is concentrated between the Benign and Recall classes.

**Fig 6:**
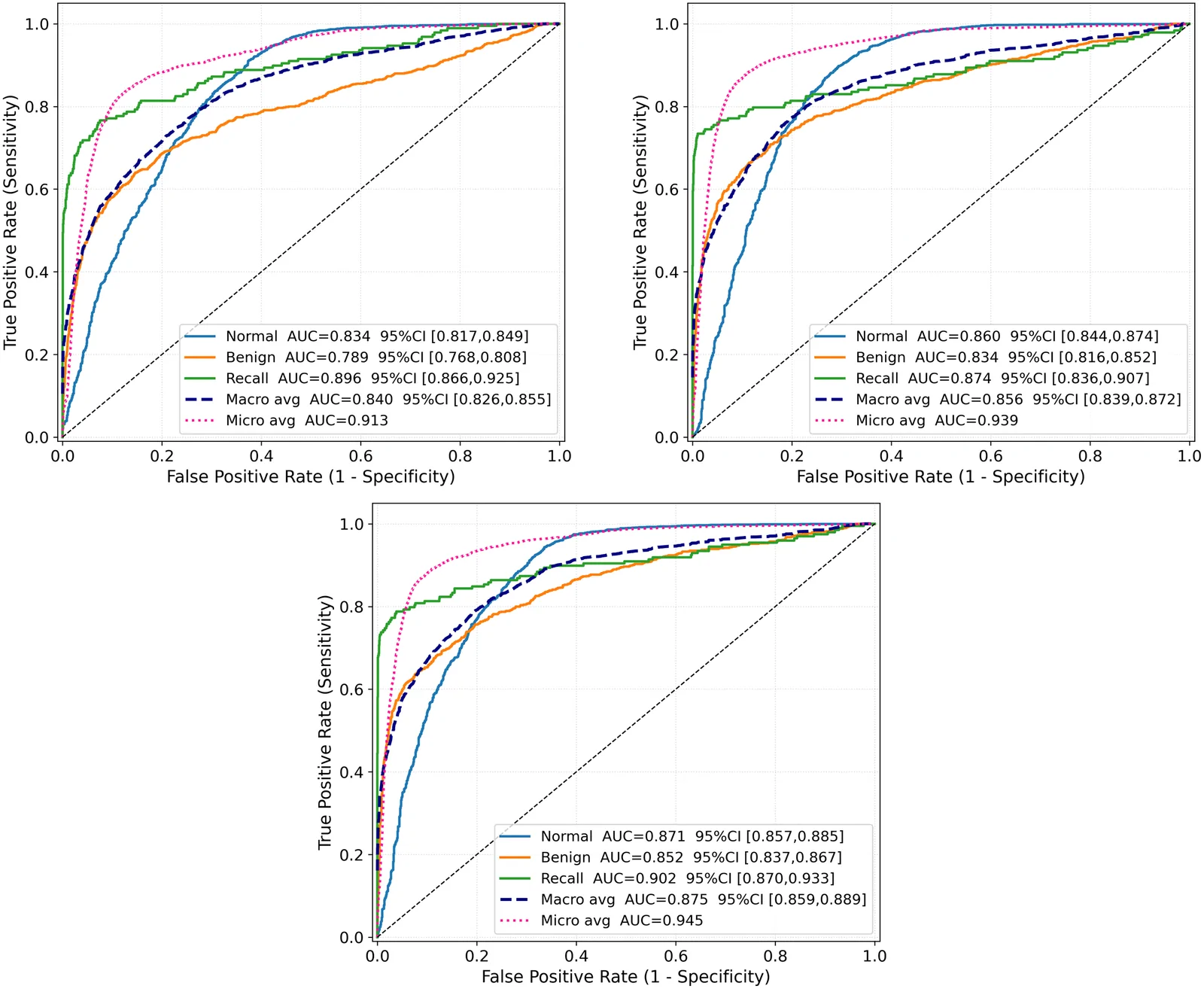
One-vs-rest ROC curves for LENS variants. The first row presents LENS-Tiny (left) and LENS-Small (right); the second row presents LENS-Base. The baseline shows the best overall class separability, with consistently higher ROC curves across most false positive rates.

The F1-vs-FLOPs trade-off plot in Fig. 4 strengthens the same interpretation. Overall, the LENS family occupies the strongest performance-efficiency region in the plot: LENS-S reaches 77.62% Macro-F1 at 31.51 GFLOPs, and LENS-B reaches 79.55% Macro-F1 at 48.56 GFLOPs, compared with 75.22% Macro-F1 at 344.15 GFLOPs for Swin-Base and 69.70% at 703.85 GFLOPs for DINOv2-Base. Notably, the smallest LENS-T also reaches remarkable performance with 68.51% Macro-F1 at a modest 22.42 GFLOPs. This superior pattern also applies to GMIC with 75.5% Macro-F1 at 95.32 GFLOPs, and ConvNeXt-B with 76.5% at 320.92 GFLOPs. The plot therefore suggests that the current lesion-aware hybrid design improves class-balanced performance without entering the high-compute regime of large CNNs and Transformer baselines. Confusion matrices analysis on Fig 5 shows that all LENS variants classify the Normal class reliably, with sensitivity above 90%. The dominant residual errors are concentrated between the Benign and Recall classes rather than between normal and abnormal breasts. This pattern is consistent with the clinical difficulty posed by borderline suspicious findings, in which lesion appearance and surrounding context can overlap across the two abnormal categories. The confusion matrices also clarify the difference between model variants. LENS-Small achieves slightly higher Recall sensitivity than LENS-Base (147 cases versus 140 cases, approximately 3% diff), whereas LENS-Base recovers more Benign cases (577 versus 541, approximately 4% diff) while preserving comparable high Normal sensitivity. The stronger Benign-Recall balance of LENS-Base is consistent with its higher overall Macro-F1 and MCC in Table 4. These results suggest LENS-Base provides better class separation across all categories, particularly in reducing false positives for Recall and improving sensitivity for both Benign and Recall. The ROC curves in Fig 6 reinforce the same interpretation in the ranking space. LENS-Base dominates on one-vs-rest AUC for all three classes, reaching 87.1%, 85.2%, and 90.2% for Normal, Benign, and Recall, respectively, compared with 85.97%, 83.39%, and 87.37% for LENS-Small, and with 83.4%, 78.9%, and 89.6% for the tiny version. The Benign class remains the most difficult one-vs-rest discrimination problem for both variants, whereas Recall retains the strongest AUC. Overall, these results confirm that larger LENS variants provide better global discrimination and more stable ranking behavior, while smaller variants tend to exhibit stronger early recall at low false-positive rates.

### Ablation study

In this section, we conduct ablation studies to investigate the contributions of individual components of LENS and the impact of the key hyperparameter top-k value. We trained and evaluated each experiment with the same protocol described in the experimental settings section. Table 7 isolates the contribution of each architectural component in LENS-base. The full model, which combines multi-scale feature aggregation, the lesion-aware head, and the main classification head, achieves the highest macro-F1 of 79.63% (95% CI: 77.65–81.41). Using only the lesion-aware branch for prediction results in the largest performance drop, to 68.49% (95% CI: 66.07–70.89), suggesting that the global contextual representation from the main branch is essential and can not be replaced by the lesion-aware branch alone. Disabling multi-scale feature aggregation while keeping both heads yields a macro-F1 of 70.74% (95% CI: 68.41–73.00), indicating that scale-diverse features are critical for distinguishing morphologically variable findings across the three-class distribution. More importantly, a substantial 6.78% drop in macro-F1 score is observed when we remove the lesion-aware head from the full model, indicating the supplementary role of the proposed weakly supervised branch. These results show that all main components work synergistically, and omitting any one leads to substantial declines in classification performance.

**Table 7:** Ablation study on LENS-base. Values are reported with bootstrap 95% confidence intervals in brackets.

Multi-scale	Lesion-aware head	Main head	Macro-F1 (%)
✓	✓	✓	**79.63 [77.65, 81.41]**
✓	✓		68.49 [66.07, 70.89]
✓		✓	72.85 [70.75, 74.92]
	✓	✓	70.74 [68.41, 73.00]

Table 8 presents the effect of the top k value across two window sizes. When the window size is set to 8, the macro F1 score reaches its optimum as k increases from 1 to 8. However, the score gradually declines as k is further increased to 16 and 32. A similar pattern is observed when the window size is reduced to 4. These results confirm that performance depends on the proposed evidence-selection mechanism.

**Table 8:** The impact of the top-*k* value with two local attention window sizes (*w* = 4 and *w* = 8). Values are reported with 95% bootstrap confidence intervals.

	Window *w* = 4	Window *w* = 8
*k*	Macro-F1 (%)	Macro-F1 (%)
1	76.57 [74.49, 78.55]	77.32 [75.36, 79.14]
8	**78.54 [76.50, 80.57]**	**79.63 [77.69, 81.55]**
16	75.96 [73.87, 77.99]	75.98 [73.91, 78.02]
32	75.74 [73.71, 77.56]	75.38 [73.24, 77.42]

### Quantitative localization evaluation

To quantitatively validate whether the lesion-aware branch identifies clinically relevant abnormalities, we evaluated its lesion-token maps against radiologist bounding-box annotations on 193 annotated test mammograms containing 269 GT boxes. As shown in Table 9, LENS achieved 73.1% Pointing Game accuracy and 81.9% Top-8 Hit Rate, demonstrating that its selected lesion tokens frequently align with clinically annotated findings. The corresponding Top-8 IoU was 0.245. Although this overlap is moderate, LENS is trained using image-level supervision and selects a sparse set of discriminative tokens rather than predicting the complete lesion bounding box. The Heatmap Coverage reached 83.1%, indicating that the lesion-score map generally produced elevated responses within clinically annotated regions.

**Table 9:** Quantitative lesion localization evaluation of LENS-Base on the annotated test subset. Metrics were computed using GT lesion bounding boxes.

Group	N	Pointing Acc (%)	Top-8 Hit (%)	Top-8 IoU	Heatmap Coverage (%)
All	193	73.1	81.9	0.245	83.1
Architectural distortion	14	42.9	64.3	0.150	71.8
Asymmetry	2	50.0	50.0	0.147	57.1
Focal Asymmetry	19	57.9	73.7	0.180	82.6
Mass	103	73.8	81.6	0.273	87.2
Suspicious calcification	40	87.5	92.5	0.244	85.9
Other	15	78.6	85.7	0.117	62.7
Correct prediction	140	86.5	92.2	0.297	84.6
Incorrect prediction	53	36.5	53.8	0.103	79.2

The finding-specific analysis further demonstrates that the highlighted regions correspond to clinically meaningful abnormalities rather than arbitrary image structures. Localization was strongest for suspicious calcifications, for which LENS achieved a Pointing Game accuracy of 87.5% and a Top-8 Hit Rate of 92.5%. Strong localization was also observed for masses, with corresponding values of 73.8% and 81.6%. By contrast, performance was lower for architectural distortion and asymmetry subgroups.

Localization performance was also markedly associated with classification correctness. For correctly classified images, Pointing Game accuracy and Top-8 Hit Rate reached 86.5% and 92.2%, respectively, compared with 36.5% and 53.8% for incorrectly classified images. Similarly, Top-8 IoU increased from 0.103 for incorrect predictions to 0.297 for correct predictions, while Heatmap Coverage increased from 79.2% to 84.6%. These results indicate that correct predictions are more frequently accompanied by spatial evidence aligned with radiologist-annotated abnormalities, whereas classification errors are associated with weaker, incomplete, or misplaced lesion prioritization.

### Lesion-aware inference examples

**Fig 7:**
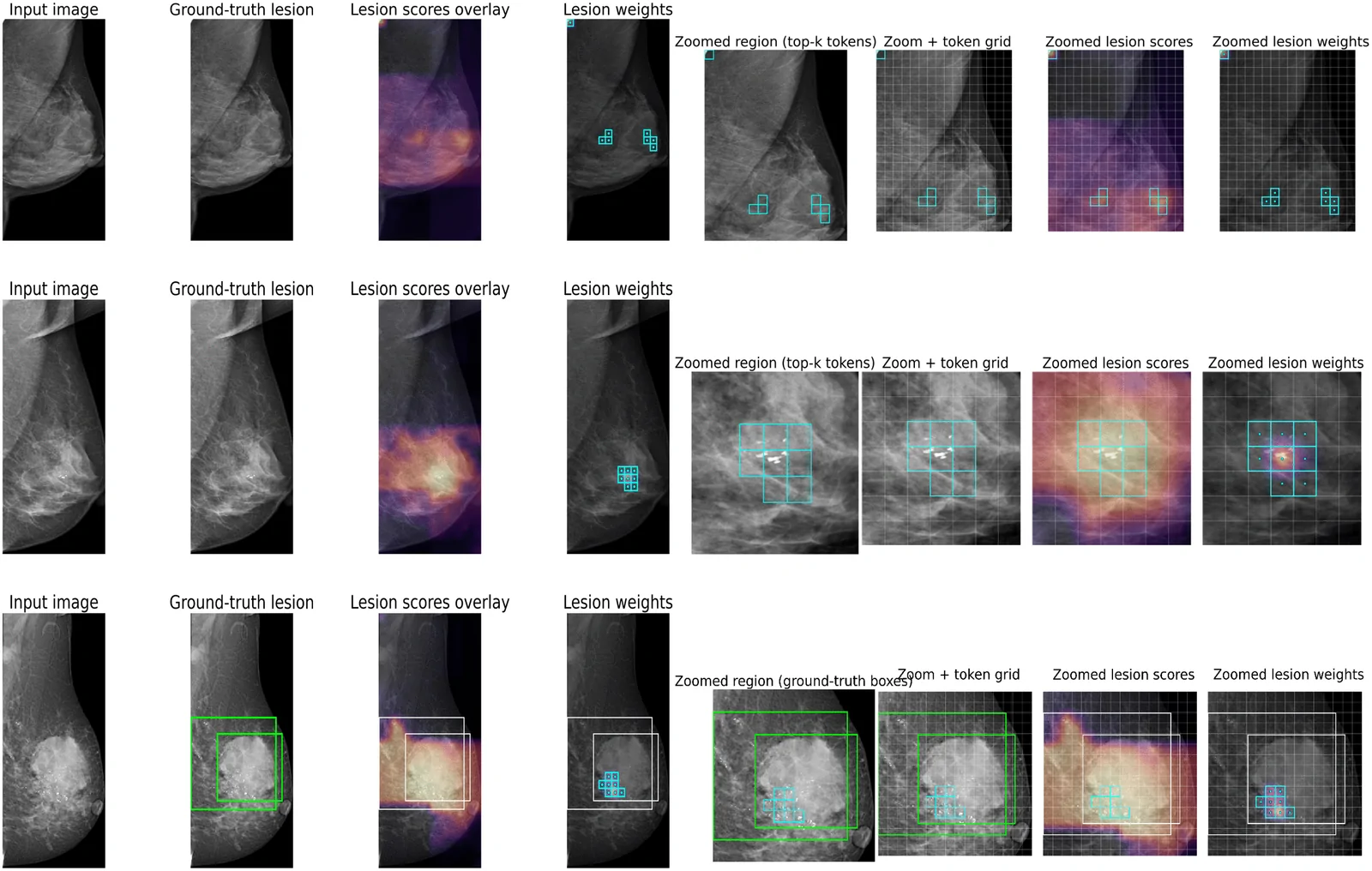
Qualitative examples of correct LENS-Base predictions. Each representative case (row) is shown with a whole-image view on the left and the corresponding zoom view on the right. Across the three classes, the lesion-aware branch remains diffuse for Normal tissue while concentrating on compact suspicious regions for Benign and Recall cases.

**Fig 8:**
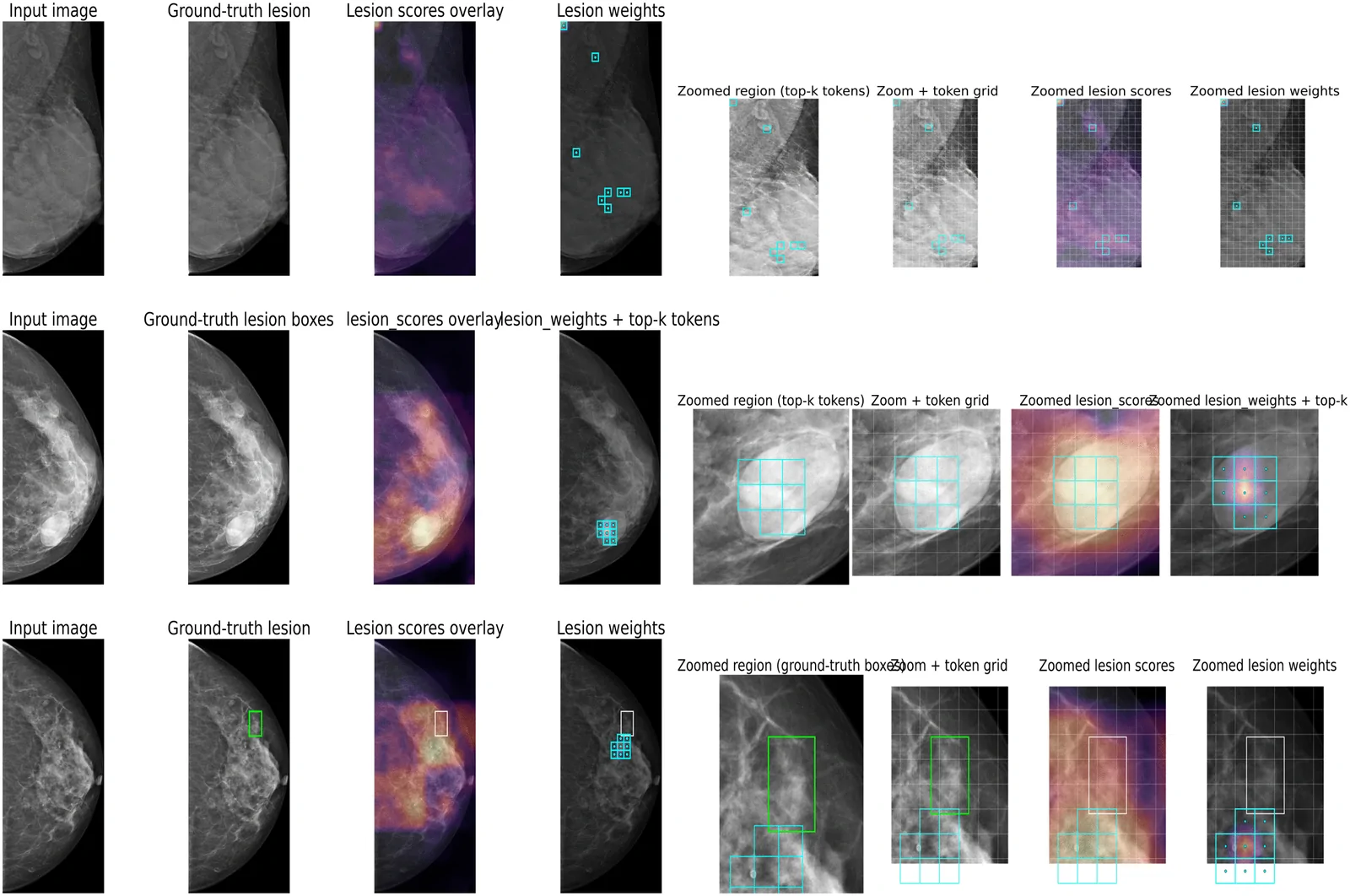
Qualitative examples of misclassification by LENS-Base. Each representative case is shown with a whole-image top view on the left and the corresponding zoom view on the right. The examples illustrate the main residual failure modes observed in the confusion matrix and localization analysis, including under-calling subtle benign findings, over-calling benign lesions as Recall, and ambiguity between Benign and Recall.

The qualitative correct-prediction examples in Fig 7 show that LENS-Base handles all three classes with visually plausible evidence routing in whole-image and zoomed views. In the Normal case (first row), the response remains diffuse and does not collapse into a spurious hotspot. This matches the absence of suspicious localized findings. In the correct Benign and Recall examples (second and third rows), the model focuses on compact regions that stay stable when zoomed around the main structure. This suggests that the lesion-aware branch improves metrics and produces class decisions from localized evidence rather than unrelated background. The misclassified examples in Fig 8 provide a more detailed error analysis than the confusion matrices. In Case 1 (first row), the model predicts Normal, while the ground truth is Benign. This outcome indicates that subtle, low-contrast abnormalities may be under-weighted when localized evidence is weak in both whole-image and zoomed views. The Benign → Recall (incorrect recall classification) in the second row and Recall → Benign (incorrect benign classification) in the third row illustrate the opposite failure mode. Even when the model attends to the relevant region, ambiguity in lesion morphology and parenchymal context can shift the final decision between the two abnormal categories. These qualitative examples are consistent with the class-level metrics: the primary challenge is not distinguishing normal from abnormal, but rather achieving fine-grained discrimination between subtle or borderline abnormal patterns.

## Discussion

### Interpretation of main findings

Mammography screening generates a large and recurring volume of examinations, placing a substantial workload on radiologists and motivating the development of robust and efficient AI-assisted CAD systems. However, the clinical adoption of AI-assisted CAD systems remains limited by concerns regarding their black-box decision-making [57]. This highlights the need for models that not only achieve strong predictive performance but also provide interpretable evidence for clinicians to examine. This study introduces LENS, a mammography-specific hybrid CNN-Transformer framework designed to provide accurate and lesion-aware classification with a competitive performance-computation trade-off. The principal findings of this study can be summarized in two complementary aspects:

First, the comprehensive comparison on the VinDr-Mammo test set showed that LENS-B achieved the strongest class-balanced performance among the evaluated architectures, as reflected by the significant improvement in Macro-F1 and competitive overall Accuracy and MCC. Its principal class-specific advantage was observed for the Recall category. It maintained competitive sensitivity while achieving the highest Recall specificity and PPV. It also achieved the highest Recall sensitivity at a fixed false-positive rate of 10%. These results indicate that LENS-B provides a favorable trade-off between identifying examinations that require further assessment and limiting unnecessary Recall predictions.

This performance profile may be attributable to the complementary design of LENS. Mammographic abnormalities are often spatially sparse, low-contrast, and embedded within large regions of normal tissue; therefore, their recognition requires both sensitivity to subtle local patterns and the ability to interpret these patterns within a broader anatomical context. The rectangular and multi-scale tokenization strategy is particularly relevant to mammography. Unlike conventional Transformers that operate on fixed square token grids, it is better aligned with the elongated geometry of mammograms and may preserve spatial relationships between localized tissue abnormalities and their surrounding breast anatomy before global contextual integration. The multi-scale convolutional components can capture fine-grained local texture and boundary information, whereas the local-global Transformer components can model longer-range dependencies across the breast region. Their combination may therefore allow subtle lesion-related features to be interpreted together with the surrounding anatomical context rather than relying exclusively on either local convolutional processing or global self-attention. Furthermore, the ablation results suggest that removing either multi-scale feature fusion, global context modeling, or lesion-focused evidence routing reduced performance. This finding indicates that the advantage of LENS arises from the complementary interaction among these components rather than from model scaling alone.

From a computational perspective, LENS provides a favorable balance between predictive performance and model complexity. LENS-B achieves substantially lower computational complexity and higher throughput than most CNN and Transformer baselines with comparable or larger model sizes. The consistent efficiency of the smaller LENS variants further indicates that the proposed architecture can be scaled according to available computational resources. Together with the ablation findings, these results suggest that the predictive gains arise primarily from the complementary integration of local, global, and lesion-focused representations rather than from increased model capacity alone.

Second, the quantitative and qualitative analyses demonstrate that the lesion-aware branch provides clinically meaningful evidence localization under image-level supervision. On the annotated test subset, LENS-Base achieved a Pointing Game accuracy of 73.1%, a Top-8 Hit Rate of 81.9%, a Top-8 IoU of 0.245, and a Heatmap Coverage of 83.1%, indicating that the highest-scoring and selected lesion tokens frequently overlap with radiologist-annotated lesions. Localization was particularly strong for suspicious calcifications and masses, whereas lower performance on architectural distortion and asymmetry may reflect the more diffuse and poorly circumscribed appearance of these findings, making them more difficult to represent using a small set of discrete tokens. Because some subgroups, particularly asymmetry, contain very few samples, these finding-specific results should be interpreted descriptively rather than as definitive comparisons between abnormality categories. Localization was markedly stronger in correctly classified cases than in incorrectly classified cases, with Pointing Game accuracy increasing from 36.5% to 86.5% and Top-8 Hit Rate from 53.8% to 92.2%. This association suggests that successful predictions are generally supported by spatial evidence aligned with clinically relevant regions. The qualitative examples in Figs 7 and 8 complement the quantitative localization results by illustrating successful evidence routing and representative failure modes. These examples clarify how the evidence routing operates across classes. For correctly classified Normal cases, the response remains diffuse without collapsing into an isolated hotspot, which is consistent with the absence of a localized suspicious finding. For correctly classified Benign and Recall cases, the model concentrates on compact regions that remain stable in zoomed views, supporting the interpretation that abnormal predictions are informed by localized lesion evidence.

The failure cases reveal two limitations. First, subtle or low-contrast abnormalities may be under-weighted when the lesion response is weak, leading to Benign cases being classified as Normal. Second, even when the model attends to the relevant region, overlap in lesion morphology and surrounding parenchymal context can shift the decision between Benign and Recall. Taken together, the observed error patterns suggest that a major remaining challenge lies in fine-grained discrimination between subtle or borderline abnormal patterns, particularly between Benign and Recall cases, although weak lesion evidence can also lead to errors in normal/abnormal separation. Nevertheless, these results should be interpreted as evidence of clinically meaningful region prioritization rather than precise lesion delineation, since only box-level annotations are available and the model predicts token-level suspicious regions rather than segmentation masks.

### Limitations and future work

This study has limitations that should be prioritized in future work. First, all experiments were conducted on a single dataset. Although the dataset includes heterogeneous acquisition settings, evaluation on a single benchmark does not establish external validity across institutions, scanner vendors, patient populations, or acquisition protocols. Because mammography performance can be sensitive to domain shift, an independent multi-center evaluation is necessary before stronger claims about generalizability can be made. Second, substantial class imbalance remains a limitation, as reflected by the lower performance observed for minority abnormal categories. Future work will investigate complementary data-level and sampling-level strategies, including realistic lesion synthesis [47] and class-aware batch sampling. Third, although all evaluated baselines were retrained under identical experimental conditions to ensure a fair comparison, the proposed framework has not yet been experimentally compared with several recently published architectures [35,36]. Future work will include these methods under the same unified evaluation protocol to provide a broader assessment of comparative predictive performance and computational trade-offs. Fourth, although the lesion-token maps showed quantitative agreement with radiologist-provided bounding boxes, this evaluation was limited to the annotated abnormal subset and used coarse box-level annotations. The results therefore support clinically relevant region prioritization but do not establish pixel-accurate lesion delineation, causal reliance on the highlighted regions, or robustness against shortcut features. Future work should incorporate external localization cohorts, reader-based assessment, and controlled lesion or background perturbation experiments.

## Conclusion

In conclusion, this study presents LENS, a mammography-specific hybrid CNN-Transformer framework designed to support lesion-focused evidence learning under image-level supervision. Quantitative localization results and qualitative visualizations showed that the lesion token responses frequently aligned with radiologist-annotated abnormalities, providing evidence that the model captures clinically relevant image regions. On the VinDr-Mammo dataset, LENS-B achieved the highest Accuracy, Macro-F1, and MCC among the evaluated models, with statistically significant improvements in Macro-F1 over all baselines. Its main class-specific advantage was observed for the clinically important Recall category, where it maintained competitive sensitivity while achieving the highest specificity, PPV, and sensitivity at a fixed 10% false-positive rate. Together with its competitive computational efficiency, the results support lesion-aware hybrid CNN-Transformer modeling as a promising direction for mammography screening AI. Nevertheless, external validation on independent datasets and prospective clinical evaluation are necessary before deployment in real-world screening workflows.
